# Supplementary material for: Understanding Social-Ecological Challenges of a Small-Scale Hilsa (Tenualosa ilisha) Fishery in Bangladesh
Source: Int J Environ Res Public Health. 2019 Nov 29;16(23):4814. doi: 10.3390/ijerph16234814 (PMC6927010; doi:10.3390/ijerph16234814)
Supplement: Supplementary file 1 [file ijerph-16-04814-s001.pdf]

<u>Semi-structured questionnaires for in-depth interviews/ Focus group discussions (FGD)</u>

Hilsa fishers (Man and woman)-

- **Background Information**- Name, age, contact number, education, family members, village name, etc.
- **Fishing and fish behavior-** What is the primary purpose of your involvement in fishing? Fishing as a profession (how long)? Do you have your own boat? Otherwise how you will manage to fish? Where do you go for Hilsa fishing? Do you know when Hilsa breed? Do you have any idea about the Hilsa fish breeding ground? Who fish there and are there any outside fishers? Do you take loan to operate your fishing activities and from whom you take loan? How you will repay the loan? Is there any agreement between you and loan giver? What are the difficulties you face during your fishing? Is Hilsa overexploited? Is jatka overexploited? Could you tell me the reasons behind this? What is your advice to minimize the situation?
- **Economy of the family**- What is your average monthly income from fishing? What are your other sources of income of the household? Are you a member of a fisher's association? If yes, then why? Are you happy with your living conditions?
- **Local ecological knowledge** - How was the fishery during your parents and grandparent's generation? What kind of gear they used, what species did they fish (Hilsa and others), were there any fishing rules? What gear types and vessels are used in the fishery? How does Hilsa vary with season and from year to year (Average catches per fishing trip, fish size, presence of eggs, year to year decrease or increase)? With whom you share your fishing experiences? How will all the knowledge be passed on to younger generations (The question will be asked to give an indication of a potential changes in traditions, knowledge transfer)?
- **Fishing rights and regulations**- Are you aware of any Hilsa management regimes introduced by the Government (Hilsa sanctuaries, jatka fishing ban period, and conservation of gravid/full of eggs Hilsa?)

- **Ban period and role of incentives-** What is a ban period of Hilsa? Do you know the importance of Hilsa ban period? When it starts? What are the rules and regulations during the Hilsa fishing ban period? How long the ban period is? Do you notice any enforcement activity take place during the ban period? Are there any poachers? What kinds of punishment are subjected to? Do you think ban period is good for you or not? What do you want to do during the ban period? How you will survive or compensate? Do you get any subsidies from the government? Is it enough for your family? Do you have new ideas to improve the situation during the

ban period? Has not being allowed to catch Hilsa in particular zones during the ban period changed your fishing behavior? If so, has this had an effect on your household income and how much? How much in household income do you get from other activities that you do during the ban period, which you wouldn't have done if you had been fishing ? If you are affected by the current ban period (or if you were to be affected in the future) how much is your minimum willingness to accept compensation in Taka?

- **Impacts of driving forces, pressures and state changes in terms of hilsa fishery stakeholder's well-being** - How climatic shocks and stresses impact your household (negatively or positively) both inland and at sea? (Shocks-unexpected and stresses-mental or emotional that include cyclones, floods, sea level changes, land erosion, and variations in temperature and rainfall, salinity intrusion, climate change and others). Furthermore, could you explain the impacts of those shocks and stresses include livelihood activities and strategies (fisheries or non-fisheries related). How you tackle these, both in the short-term (during the time when it happens) and in the longer-term (after it happens and within few months)? What things facilitate or constrain while tackling these? What things help most while tackling these and how? Describe the major socio-economic and environmental concerns you face in your livelihoods and the fishing communities? Do you think environmental changes or climate changes over time impacted on production of fish and your catch? Do you see any species of fish is no more available that you used to catch them before? In your opinion what are the reasons behind losing fish species? Please, specify? What is your overall experience about environmental change over time in this area and influence on your livelihoods? Is your livelihood affected by the closed/off season and zone? How enough is the compensation provided? If you are not a recipient or the compensation is not enough, what are the most important strategies you do to cope in times of hardship?
- **Possible responses to increase the sustainability of the hilsa fishery -** What are the problems of fisheries management? Suggest actions and measures that should be taken to alleviate problems in the livelihoods of fishers, and the fishing communities. Do you think the compensation scheme has had positive impact on Hilsa stock regeneration, Hilsa catch levels and the improvement of fisher's livelihoods? Over the last years, do you think Hilsa stocks have changed as a direct result of the compensation scheme? If no change, what is the reason? Do you think the distribution of compensation is fair, why and why not? Which people in the village get compensation? Who do you think should be receiving compensation? In your opinion, what is the level of compliance with the ban period and If not, everyone complies in a group, then (in your opinion) what needs to be done to enhance compliance within these groups? Why do you think the pressure

to repay loan is an obstruction to abide with ban period? Do you think the fishing ban period fits well with the Hilsa breeding season? Do you know something about co-management? Are you involved in fisheries management and co-management, please explain what advantages and disadvantages of the co-management system are? Who is involved in the co-management processes and what is their role? What co-management activities have been carried out in your village or areas? Do you have ideas, who is funding for the co-management activities? What do you think about the role of the local leaders and government officials in developing and implementing co-management? What do you think about the power of fishers in co-management in your village/union/upzilla? What factors affecting the exercise of fishers' powers? What do you think about the government policies and legal support for co-management in your village? What are the most significant changes in your village after co-management initiated? In your opinion what are the shortcoming of the co-management system? What is your advice / are your suggestions for successful co-management in your locality? In your opinion, what are the conditions for you as an individual to continue participating in co-management activities? Do you think that the co-management activities would be continued without any external support and how you will do that?

Specific questions to other stakeholders involved in hilsa fishery:

- **Boat owners**: How you are involved in Hilsa fishery? What are the conflicts/cooperation that you have with other stakeholders?
- **Fish traders**: Are you a currently a fisher? Were you a fisher in the past? Since when local fishers started to sell their catches to you? When did you start to sell fishing products? What fishing products sell the best? Do you sell only Hilsa fish? Do you have any arrangements with any of the local fishers to have them sell their products exclusively to you? What is this arrangement? How many fishers only fish for you?
- **Money lenders**: What is the primary purpose of your involvement in fishing? What do you think about the co-management arrangements? How do you think you can co-operate in the co-management arrangement? Do you think that you need compensation as well during the ban period? What are the possible responses to increase the sustainability of the hilsa fishery, in your opinion?
- **Local NGOs** (officials working with fishing communities): What is the primary purpose of your involvement in Hilsa fishing? What do you think about the co-management arrangements? How do you think you can co-operate in the co-management arrangement?
- **Local government representatives** (Upazila Chairman, Union Parishad Chairman, Union Parishad Member)- What is the primary purpose of your involvement in fishing? What types of problem do you face while implementing the government fisheries regulations? What are the essential things that you consider implementing the incentive scheme among hilsa fishers?
- **Local governments administrative personnel's (Fishery officer, Police and Coast Guard)**: What is the primary purpose of your involvement in fishing? What types of problem you face while implementing the government fisheries regulations? What is your

idea about the comanaging Hilsa fishery? What do you think about the ban period and its outcomes?

- **Academics**- How you are involved in Hilsa fishery? In your opinion, what are the driving forces and pressures you found in the Hilsa fishery? What are the state changes in the hilsa fishery due to driving forces and pressures? What are the impacts of driving forces, pressures and state changes in terms of hilsa fishery stakeholder's well-being? Please suggest actions and measures that should be taken to alleviate problems in the livelihoods of fishers, and the fishing communities.
- **Environmental Specialists**- How you are involved in Hilsa fishery? In your opinion, what are the driving forces and pressures you found in the Hilsa fishery? What are the state changes in the hilsa fishery due to driving forces and pressures? What are the impacts of driving forces, pressures and state changes in terms of hilsa fishery stakeholder's well-being? Please suggest actions and measures that should be taken to alleviate problems in the livelihoods of fishers, and the fishing communities.
- **Aquaculture Specialists**- How you are involved in Hilsa fishery? In your opinion, what are the driving forces and pressures you found in the Hilsa fishery? What are the state changes in the hilsa fishery due to driving forces and pressures? What are the impacts of driving forces, pressures and state changes in terms of hilsa fishery stakeholder's well-being? Please suggest actions and measures that should be taken to alleviate problems in the livelihoods of fishers, and the fishing communities. Is it possible to do aquaculture of Hilsa? If yes, How and what types of research is necessary in Bangladesh?

Table S1: The scientific literature related to the hilsa fishery management in Bangladesh from 2001-2019

No	Article
1	Hossain, M. A., Das, I., Genevier, L., Hazra, S., Rahman, M., Barange, M., & Fernandes, J. A. (2019). Biology and fisheries of Hilsa shad in Bay of Bengal. *Science of the Total Environment*, *651*, 1720-1734.
2	Hossain, M. S., Sharifuzzaman, S. M., Rouf, M. A., Pomeroy, R. S., Hossain, M. D., Chowdhury, S. R., & AftabUddin, S. (2019). Tropical hilsa shad (Tenualosa ilisha): Biology, fishery and management. *Fish and Fisheries*, *20*(1), 44-65.
3	Sahoo, A. K., Wahab, M. A., Phillips, M., Rahman, A., Padiyar, A., Puvanendran, V., ... & Behera, B. K. (2018). Breeding and culture status of Hilsa (Tenualosa ilisha, Ham. 1822) in South Asia: a review. *Reviews in Aquaculture*, *10*(1), 96-110.
4	Islam, M., Aktar, R., Nahiduzzaman, M., Barman, B., & Wahab, M. (2018). Social considerations of large river sanctuaries: a case study from the Hilsa shad fishery in Bangladesh. *Sustainability*, *10*(4), 1254.
5	van Brakel, M. L., M. Nahiduzzaman, A. Mahfuzul Haque, M. Golam Mustafa, M. Jalilur Rahman, and M. Abdul Wahab. 2018. Reimagining large-scale open-water fisheries governance through adaptive comanagement in hilsa shad sanctuaries. *Ecology and Society* 23(1):26
6	Mozumder, M., Wahab, M., Sarkki, S., Schneider, P., & Islam, M. (2018). Enhancing social resilience of the coastal fishing communities: A case study of hilsa (Tenualosa ilisha H.) fishery in Bangladesh. *Sustainability*, *10*(10), 3501.
7	Hossain, A. A., Bisshas, S., Pramanik, M. M. H., Hasan, M. M., Haidar, M. I., Bosu, A., ... & Rahman, M. A. (2018). Supply Chain Analysis of Hilsa (Tenualosa Ilisha) Egg in Bangladesh. *Journal of FisheriesSciences. com*, *12*(4), 9-12.
8	Islam, M. M., & Chuenpagdee, R. (2018). Nomadic Fishers in the Hilsa Sanctuary of Bangladesh: The Importance of Social and Cultural Values for Wellbeing and Sustainability. In *Social Wellbeing and the Values of Small-scale Fisheries* (pp. 195-216). Springer, Cham.
9	Debnath, S., Latifa, G. A., Bhowmik, S., Islam, S., & Begum, M. (2018). Comparative analysis of nutritional values of riverine and marine hilsa (Tenualosa ilisha; Hamilton, 1882). *Korean Journal of Agricultural Science*, *45*(2), 258-264.
10	Porras, I., Mohammed, E. Y., Ali, L., Ali, M. S., & Hossain, M. B. (2017). Power, profits and payments for ecosystem services in Hilsa fisheries in Bangladesh: a value chain analysis. *Marine Policy*, *84*, 60-68.
11	Jahan, I., Ahsan, D., & Farque, M. H. (2017). Fishers' local knowledge on impact of climate change and anthropogenic interferences on Hilsa fishery in South Asia: evidence from Bangladesh. *Environment, development and sustainability*, *19*(2), 461-478.
12	Rahman, M. A., Ahmed, T., Pramanik, M. M. H., & Flura, H. M. (2017). On-board Breeding Trial of Hilsa (Tenualosa ilisha, Ham. 1822) and Testing of Larval Rearing in Bangladesh. *J Aquac Res Development*, *8*(471), 2.
13	Pramanik, M. M. H., Rahman, M. A., Ahmed, T., & Flura, H. M. (2017). Gill net selectivity of hilsa (Tenualosa ilisha) in the Meghna River Estuary of Bangladesh. *J Aquac Res Development*, *8*(483), 2.
14	Hossain, M. S. (2017). The shifting habitat of Hilsa: River to sea. Secretariat, Southeast Asian Fisheries Development Center.
15	Bala, B. K., Arshad, F. M., & Noh, K. M. (2017). Modelling of Hilsa Fish (Tenualosa ilisha) Population in Bangladesh. In *System Dynamics* (pp. 179-198). Springer, Singapore.
16	Rahman, M. A., Ahmed, T., Pramanik, M. M. H., & Flura, H. M. (2017). On-board Breeding Trial of Hilsa (Tenualosa ilisha, Ham. 1822) and Testing of Larval Rearing in Bangladesh. *J Aquac Res Development*, *8*(471), 2.
17	Rahman, M. A., Pramanik, M. M. H., Flura, A. T., & Hasan, M. M. (2017). Impact Assessment of Twenty-Two Days Fishing Ban in the Major Spawning Grounds of Tenualosa

	ilisha (Hamilton, 1822) on its Spawning Success in Bangladesh. *J Aquac Res Develop*, *8*, 489.
18	Islam et al., 2016. Economic incentives for sustainable hilsa fishing in Bangladesh: An analysis of the legal and institutional framework. *Marine policy*, *68*, 8-22.
19	Bladon, A. J., Short, K. M., Mohammed, E. Y., & Milner-Gulland, E. J. (2016). Payments for ecosystem services in developing world fisheries. *Fish and fisheries*, *17*(3), 839-859.
20	Islam, M. M., Islam, N., Sunny, A. R., Jentoft, S., Ullah, M. H., & Sharifuzzaman, S. M. (2016). Fishers' perceptions of the performance of hilsa shad (Tenualosa ilisha) sanctuaries in Bangladesh. *Ocean & coastal management*, *130*, 309-316.
21	Hasan, K. M. M., Ahmed, Z. F., Wahab, M. A., & Mohammed, E. Y. (2016). *Food and feeding ecology of hilsa (Tenualosa ilisha) in Bangladesh's Meghna River basin* (p. 20). IIED Working Paper. IIED, London.
22	Hossain, M. S., Sharifuzzaman, S. M., Chowdhury, S. R., & Sarker, S. (2016). Habitats across the life cycle of hilsa shad (Tenualosa ilisha) in aquatic ecosystem of Bangladesh. *Fisheries Management and Ecology*, *23*(6), 450-462.
23	Islam, M. M., Shamsuzzaman, M. M., Sunny, A. R., & Islam, N. (2016). Understanding fishery conflicts in hilsa sanctuaries of Bangladesh. *Inter-Sectoral Governance of Inland Fisheries; Song, AM, Bower, SD, Onyango, P., Cooke, SJ, Chunepagdee, R., Eds*.
24	Faruque, H., Ahsan, D. A., Sarker, M. H., & Gladun, E. F. (2016). Effect of Ganges river morphological dynamics and Farakka barrage on upward migration and catch of Indian shad (Tenualosa ilisha) in Bangladesh. Tyumen State University Herald. *Natural Resource Use and Ecology*, *2*(3), 34-58.
25	Bhaumik, U. (2016). Stock profile of Hilsa Shad population in Bay of Bengal region-a review. *Int. J. Curr. Res. Aca. Rev*, *4*(6), 22-38.
26	Md, S. J., Uddin, A. M. M. B., Md, P. S., Tanmay, M. H., & Rahman, F. (2016). Livelihood status of Hilsa (Tenualosa ilisha) fishermen of greater Noakhali regions of Bangladesh. *Fish Aquac J*, *7*(168), 2.
27	Mohammed, E. Y., Ali, L., Ali, S., Hussein, B., Wahab, M. A., & Sage, N. (2016). Hilsa's non-consumptive value in Bangladesh: estimating the non-consumptive value of the hilsa fishery in Bangladesh using the contingent valuation method. *International Institute for Environment and Development (IIED) Working Paper. IIED, London, UK. [online] URL: http://pubs. iied. org/16626IIED*.
28	Bladon, A., Hassan, S. A., Uddin, A. T., Ali, N., Ali, L., Hussein, S. B., ... & Steele, P. (2016). Finding evidence for the impact of incentive-based Hilsa fishery management in Bangladesh: combining theory testing and remote sensing methods. *International Institute for Environment and Development, Working Paper, London*.
29	Begum, M., Bhowmik, S., Juliana, F. M., & Hossain, M. S. (2016). Nutritional Profile of Hilsa Fish [Tenualosa ilisha (Hamilton, 1822)] in Six Selected Regions of Bangladesh. *J. Nutr. Food Sci*, *6*(2).
30	Miah, M. S. (2015). Climatic and anthropogenic factors changing spawning pattern and production zone of Hilsa fishery in the Bay of Bengal. *Weather and Climate Extremes*, *7*, 109-115.
31	Flura, M. Z., Rahman, B. S., Rahman, M. A., Ashraful, M., Alam, M., & Pramanik, M. H. (2015). Length-weight relationship and GSI of hilsa, Tenualosa ilisha (hamilton, 1822) fishes in Meghna river, Bangladesh. *International Journal of Natural and Social Sci*, *2*(3), 82-88.
32	Dewan, B. K., Mia, M. S., Yeasmin, F., Sarker, S. C., Siddiky, M. N., & Kamal, M. (2015). Studies on the proximate composition of Hilsa of different size groups at Chandpur region. *Int J Nat Soc Sci*, *2*(5), 52-55.
33	Rahman, M. A., Ahmed, T., Pramanik, M. M. H., & Alam, M. A. (2015). Impact of fifteen days fishing ban in the major spawning grounds of hilsa (Tenualosa ilisha Hamilton 1822) on its spawning success. *Research in Agriculture Livestock and Fisheries*, *2*(3), 491-497.
34	Toufique, K. A. (2015). Some Thoughts on Hilsa Exports and Management in Bangladesh. *Bangladesh Development Studies*, *38*(2), 115-127.

35	Karim, R., Roy, K. C., Roy, P. R., & Ahmed, Z. F. (2015). Age and growth of hilsa shad, Tenualosa ilisha (Hamilton, 1822) of the river Tentulia in Bangladesh. *Journal of Fisheries*, *3*(1), 227-232.
	Roy et al., 2015.Effects of Incentive Based Hilsa Shad (Tenualosa ilisha) Management and Conservation Strategies in Bangladesh J. Sylhet Agril. Univ. 2(1):69-77, 2015
36	Ali, M. Y., Rahmatullah, R., Asadujjaman, M., Bablu, M. G. U., & Sarwer, M. G. (2015). Impacts of banning period on the socio-economic condition of hilsa fishermen in shakhchor union of lakshmipursadarupazila, Bangladesh. *Middle-East J. Sci. Res*, *23*, 2479-2483.
37	Hossain, M. S., Sarker, S., Chowdhury, S. R., & Sharifuzzaman, S. M. (2014). Discovering spawning ground of Hilsa shad (Tenualosa ilisha) in the coastal waters of Bangladesh. *Ecological Modelling*, *282*, 59-68.
38	Bala, B. K., Arshad, F. M., Alias, E. F., Sidique, S. F., Noh, K. M., Rowshon, M. K., ... & Islam, M. M. (2014). Sustainable exploitation of hilsa fish (Tenualosa ilisha) population in Bangladesh: Modeling and policy implications. *Ecological modelling*, *283*, 19-30.
39	Hossain, M. S., Sarker, S., Sharifuzzaman, S. M., & Chowdhury, S. R. (2014). Habitat modelling of juvenile Hilsa Tenualosa ilisha (Clupeiformes) in the coastal ecosystem of the northern Bay of Bengal, Bangladesh. *Journal of ichthyology*, *54*(2), 203-213.
40	Naser, N. M. (2014). Conserving trans-boundary migratory hilsa (Tenualosa ilisha) fish: a review of Bangladesh experience. In *Rivers for life. Proceedings of the International Symposium on River Biodiversity: Ganges-Brahmaputra River System, Ecosystems for life, a Bangladesh India Initiative, IUCN, International Union for Conservation of Nature* (pp. 215-221).
41	Faruque, M. H., & Ahsan, D. A. (2014). Socio-economic status of the Hilsa (Tenualosa ilisha) fishermen of Padma river, Bangladesh. *World Appl. Sci. J*, *32*(5), 857-864.
42	Rahman, H. Z., Choudhury, L. A., & Wahab, M. A. (2014). *Hilsa and hilsa fishermen: exploring conservation-livelihood win-wins*. Power and Participation Research Centre.
43	Hossain, M. K., & Arnason, R. (2014). Toward optimal use of Bangladesh Hilsa resource: Bioeconomic modelling. *United Nations University Fisheries Training Programme, Iceland [final project]*.
44	Moniruzzaman, S. M., Mortuza, M. G., Nur-Un-Nesa, M., Alam, M. A., & Alam, A. N. (2014). Spatial variations in the nutritional profiles of young, spent, ripe or gravid hilsa (Tenualosa ilisha) flesh and eggs. *International Journal of Natural and Social Sciences*, *1*(2), 61-70.
45	Ahsan, D. (2014). Impact of Climate Change and Anthropogenic Effect on Hilsa Fishery Management in South-East Asia: Urgent Need for Trans-Boundary Policy. In *WASET Conference: ICPMSS 2014: XII International Conference on Psychology, Management and Social Science*. World Academy of Science, Engineering and Technology.
46	Islam, M., Mohammed, E. Y., & Ali, L. (2014). Economic incentives for sustainable hilsa fish management in Bangladesh. *Fisheries*.
47	Wahab, M. A., Phillips, M., & Mohammed, E. Y. (2013). 10 Payments for hilsa fish (Tenualosa ilisha) conservation in Bangladesh. *Economic incentives for marine and coastal conservation: Prospects, challenges and policy implications*, 170.
48	Mohammed, E. Y. (2013). Payments for hilsa fish (Tenualosa ilisha) conservation in Bangladesh. In *Economic Incentives for Marine and Coastal Conservation* (pp. 196-215). Routledge.
49	Bhuiyan, A. I. (2013). Endoparasitic helminths of Tenualosa ilisha in Bangladesh in relation to sex, seasons and habitat type. *Bangladesh Journal of Zoology*, *41*(2), 153-163.
50	M.A. Rahman, B.M.S. Rahman, S. J. Hasan, Flura, T. Ahmed and M. I. Haidar (2013). Impact of Eleven Days Fishing Ban in the Major Spawning Grounds of Hilsa (Tenualosa Ilisha Hamilton) on its Breeding Success. Bangladesh Res. Pub. J. 9(2): 116-122. Retrieve from http://www.bdresearchpublications.com/admin/journal/upload/1309117/1309117.pdf
51	Alam, A. K. M. N., Mohanty, B. P., Hoq, M. E., & Thilshed, S. (2012, September). Nutritional values, consumption and utilization of Hilsa Tenualosa ilisha (Hamilton 1822). In *Proc. Regional workshop on Hilsa: potential for aquaculture* (pp. 16-17).

52	Rahman, M. A., Alam, M. A., Hasan, S. J., & Jaher, M. (2012). Hilsa fishery management in Bangladesh. *Hilsa: Status of fishery and potential for aquaculture*, 40-60.
53	Rahman, M. A., Alam, M. A., Hasan, S. J., & Jaher, M. (2012, September). Hilsa fishery management in Bangladesh ‘in Anon (ed.) Hilsa: Status of Fishery and Potential for Aquaculture. In *Proceedings of the Regional Workshop held in Dhaka* (pp. 16-17).
54	Hossain, M., Adhikary, R. K., Mahbub, K. R., Begum, M., & Ul Islam, M. R. (2012). Effect of 10% concentrations of salt, garlic and coriander on the quality of smoked hilsa Fish (Tenualosa ilisha). *American Journal of Food Technology*.
55	Bhuiyan, A. I., & Momen, M. (2012). Studies on the protozoan parasites of Hilsa Shad, Tenualosa Ilisha in Bangladesh. *Bangladesh Journal of Zoology*, *40*(1), 33-41.
56	Alam, A. K. M. N., Mohanty, B. P., Hoq, M. E., & Thilshed, S. (2012, September). Nutritional values, consumption and utilization of Hilsa Tenualosa ilisha (Hamilton 1822). In *Proc. Regional workshop on Hilsa: potential for aquaculture* (pp. 16-17).
57	Ahsan, D. A., Kabir, A. N., Rahman, M. M., Mahabub, S., Yesmin, R., Faruque, M. H., & Naser, M. N. (2012). Plankton composition, abundance and diversity in Hilsa (Tenualosa ilisha) migratory rivers of Bangladesh during spawning season. *Dhaka University Journal of Biological Sciences*, *21*(2), 177-189.
58	Rahman, M. J., Rahman, M. A., & Bhaumik, U. (2012, September). Biology and ecology of hilsa shad, Tenualosa ilisha (Ham.). In *Hilsa: Status of Fishery and Potential for Aquaculture, Proceedings of the Regional Workshop held in Dhaka* (pp. 16-17).
59	Sharma, A. P., Roy, N. C., & Barman, B. K. (2012, September). Hilsa: Its social, cultural, and religious importance. In *Hilsa: status of fishery and potential for aquaculture. Proceedings of the Regional Workshop held in Dhaka* (pp. 16-17).
60	Haldar, G. C., Wahab, M. A., Puvanendran, V., & Phillips, M. J. (2012, September). Potential sources of fry and fingerlings of Hilsa for aquaculture. In *Hilsa: Status of Fishery and Potential for Aquaculture, Proceedings of the Regional Workshop held in Dhaka* (pp. 16-17).
61	Rahman, 2011. Environmental impact on hilsa (Tenualosa ilisha) fisheries in the coastal belt of Bangladesh. *Ecosystem Health and Management of Pollution in the Bay of Bengal*, 69.
62	Shamsuzzaman, M. M., Mazumder, S. K., Siddique, M. A., & Miah, M. N. U. (2011). Microbial quality of hilsa shad (Tenualosa ilisha) at different stages of processing. *Journal of the Bangladesh Agricultural University*, *9*(2), 339-344.
63	Shamim, M. A. H., Ahmed, M. K., & Abdullah, A. T. M. (2011). Proximate composition of different portion of hilsa, Tenualosa ilisha from two regions of the Bay of Bengal in Bangladesh. *Dhaka University Journal of Biological Sciences*, *20*(2), 109-115.
64	Milton, D. A. (2010). Status of Hilsa (Tenualosa ilisha) management in the Bay of Bengal: an assessment of population risk and data gaps for more effective regional management.
65	Dey, S. C., Sarker, B. S., Saha, D., & Adhikary, R. K. (2010). *Impacts of Banning Period on the Socio-Economic Condition of Hilsa Fishermen of Monpura Island, Bangladesh* (Doctoral dissertation, MS Thesis, Department of Fisheries And Marine Science, NSTU, Noakhali).
66	Mazumder, S. K., & Alam, M. (2009). High levels of genetic variability and differentiation in hilsa shad, Tenualosa ilisha (Clupeidae, Clupeiformes) populations revealed by PCR-RFLP analysis of the mitochondrial DNA D-loop region. *Genetics and molecular biology*, *32*(1), 190-196.
67	Bhuiyan, A. I., D’SILVA, J., & Bristow, G. A. (2009). Parasites of Hilsa shad, Tenualosa ilisha in Bangladesh. *Bangladesh J. Zool*, *37*(2), 221-230.
68	Rahman, M. A., Alam, M. A., Ahmed, T., Ahmed, K. K. U., & Haldar, G. C. (2009). Assessment of impact of ten days fishing ban in the major spawning grounds of hilsa (Tenualosa ilisha, Fisher and Bianchi, 1984). *Bangladesh Journal of Fisheries Research*, *13*(1), 27-33.
69	Ahmed et al ., 2008. Age, growth and mortality of hilsa shad, Tenualosa ilisha in the River Meghna, Bangladesh. *Asian J. Biol. Sci*, *1*, 69-76.
70	Amin et al., 2008. Catch per unit effort, exploitation level and production of Hilsa Shad in Bangladesh waters. *Asian Fish. Sci*, *21*(175), 37.

71	Mome and Arnason, 2008. The Artisanal Hilsa Fishery: economically Efficient Fisheries Policy. IIFET 2008 Vietnam Proceedings.
72	Rahman and Cowx, 2008. Population dynamics of hilsa shad (Tenualosa ilisha, Clupeidae) in Bangladesh waters. *Asian Fisheries Science*, *21*, 85-100.
73	Mondal et al., 2008. Relationships between Fecundity and Total Length, Body Weight, Ovary Length, and Ovary Weight of Hilsa Shad, Tenualosa ilisha Hamilton, in Patuakhali, Bangladesh. *Fisheries and aquatic sciences*, *11*(2), 98-102
74	Mome et al., 2007. The potential of the artisanal hilsa fishery in Bangladesh: an economically efficient fisheries policy. *Fisheries Training Programme Final Project Report, United Nations University, Iceland*, 57.
75	Mazid et al., 2007. Source and abundance of Jatka (juvenile hilsa, Tenualosa ilisha) in the Gajner beel, Sujanagar, Pabna. *Bangladesh Journal of Fisheries*, *30*, 37-51.
76	Akter, M. A., Hossain, M. D., Hossain, M. K., Afza, R., & Bhuyian, A. S. (2007). The fecundity of Hilsa ilisha from the river Padma near Godagari of Rajshahi district. *University Journal of Zoology, Rajshahi University*, *26*, 41-44.
77	Rahman, 2006. Recent advances in the biology and management of Indian shad (Tenualosa ilisha Ham.). *SAARC Journal of Agriculture*, *4*, 67-90.
78	Rahman and Cowx 2006. Lunar periodicity in growth increment formation in otoliths of hilsa shad (Tenualosa ilisha, Clupeidae) in Bangladesh waters. *Fisheries research*, *81*(2), 342-344.
79	Mazid, M. A., Rahman, M. J., & Mustafa, M. G. (2005). Abundance, migration and management of Jatka (juvenile hilsa, Tenualosa ilisha) in the Gajner Beel, Pabna, Bangladesh. *Bangladesh Journal of Fisheries Research*, *9*(2), 191-202.
80	Nurul Amin, S. M., Arshad, A., Haldar, G. C., Shohaimi, S., & Ara, R. (2005). Estimation of Size Frequency Distribution, Sex Ratio and Length-Weight Relationship of Hilsa Shad (Tenualosa ilisha) in the Bangladesh Water. *Research Journal of Agriculture and Biological Sciences*, *1*(1), 61-66.
81	Salini et al., 2004. Allozyme and morphological variation throughout the geographic range of the tropical shad, hilsa Tenualosa ilisha. *Fisheries Research*, *66*(1), 53-69.
82	Saifullah, A. S. M., Rahman, M. S., & Khan, Y. S. A. (2004). Fecundity of Hilsa His ha (Hamilton, 1822) from the Bay of Bengal. *Pakistan Journal of Biological Sciences*, *7*(8), 1394-1398.
83	Amin et al., 2004. Stock assessment and management of Tenualosa ilisha in Bangladesh. *Asian Fisheries Science*, *17*(1/2), 50-60.
84	Halder, G. C. (2004). Present status of the hilsa fishery in Bangladesh. *GC Halder//Completion Report of the Studies Conducted under the ARDMCS, GEF Component*.
85	Shifat et al., 2003. Use of RAPD fingerprinting for discriminating two populations of Hilsa shad (Tenualosa ilisha Ham.) from inland rivers of Bangladesh. *BMB Reports*, *36*(5), 462-467.
86	Blaber et al., 2003.New insights into the life history of Tenualosa ilisha and fishery implications. In *American Fisheries Society Symposium* (pp. 223-240). AMERICAN FISHERIES SOCIETY.
87	Amin et al., 2002. Population dynamics and stock assessment of Hilsa shad, Tenualosa ilisha in Bangladesh. *Asian Fisheries Science*, *15*(2), 123-128.
88	Ahmed, S., Mostafa, G., Saiful, B. R., Arafat, Y., & Ali, S. (2002). Some aspects of population dynamics of hilsa shad Tenualosa ilisha in the River Meghna of Bangladesh. *Fisheries science*, *68*(sup1), 318-321.
89	Milton, D. A., & Chenery, S. R. (2001). Can otolith chemistry detect the population structure of the shad hilsa Tenualosa ilisha? Comparison with the results of genetic and morphological studies. *Marine Ecology Progress Series*, *222*, 239-251.
90	Rahman, M. A., Haldar, G. C., Milton, D. A., Mazid, M. A., & Miah, M. S. (2001). A study on the population dynamics of hilsa, Tenualosa ilisha (Hamilton-Buchanan) in Bangladesh. *Indian Journal of Animal Sciences*, *71*(1), 71-73.
